# Development of a Machine-Learning Model for Prediction of Extubation Failure in Patients with Difficult Airways after General Anesthesia of Head, Neck, and Maxillofacial Surgeries

**DOI:** 10.3390/jcm12031066

**Published:** 2023-01-30

**Authors:** Huimin Huang, Jiayi Wang, Ying Zhu, Jinxing Liu, Ling Zhang, Wei Shi, Wenyue Hu, Yi Ding, Ren Zhou, Hong Jiang

**Affiliations:** Department of Anesthesiology, Shanghai Ninth People’s Hospital, Shanghai Jiao Tong University School of Medicine, Shanghai 200023, China

**Keywords:** postoperative difficult airway, extubation failure, machine learning, head neck and maxillofacial surgeries, general anesthesia

## Abstract

(1) Background: Extubation failure after general anesthesia is significantly associated with morbidity and mortality. The risk of a difficult airway after the general anesthesia of head, neck, and maxillofacial surgeries is significantly higher than that after general surgery, increasing the incidence of extubation failure. This study aimed to develop a multivariable prediction model based on a supervised machine-learning algorithm to predict extubation failure in adult patients after head, neck, and maxillofacial surgeries. (2) Methods: A single-center retrospective study was conducted in adult patients who underwent head, neck, and maxillofacial general anesthesia between July 2015 and July 2022 at the Shanghai Ninth People’s Hospital. The primary outcome was extubation failure after general anesthesia. The dataset was divided into training (70%) and final test sets (30%). A five-fold cross-validation was conducted in the training set to reduce bias caused by the randomly divided dataset. Clinical data related to extubation failure were collected and a stepwise logistic regression was performed to screen out the key features. Six machine-learning methods were introduced for modeling, including random forest (RF), k-nearest neighbor (KNN), logistic regression (LOG), support vector machine (SVM), extreme gradient boosting (XGB), and optical gradient boosting machine (GBM). The best performance model in the first cross-validation dataset was further optimized and the final performance was assessed using the final test set. (3) Results: In total, 89,279 patients over seven years were reviewed. Extubation failure occurred in 77 patients. Next, 186 patients with a successful extubation were screened as the control group according to the surgery type for patients with extubation failure. Based on the stepwise regression, seven variables were screened for subsequent analysis. After training, SVM and LOG models showed better prediction ability. In the k-fold dataset, the area under the curve using SVM and LOG were 0.74 (95% confidence interval, 0.55–0.93) and 0.71 (95% confidence interval, 0.59–0.82), respectively, in the k-fold dataset. (4) Conclusion: Applying our machine-learning model to predict extubation failure after general anesthesia in clinical practice might help to reduce morbidity and mortality of patients with difficult airways after head, neck, and maxillofacial surgeries.

## 1. Introduction

The perioperative management of difficult airways has always been the focus of anesthesia. In recent years, with the wide application of new technologies and methods in endotracheal intubation, the number of serious adverse events related to endotracheal intubation has significantly decreased. Meanwhile, the incidence of serious adverse events related to tracheal extubation after general anesthesia has not significantly changed [1,2]. Tracheal extubation is a key step in the recovery from general anesthesia. Head, neck, and maxillofacial surgeries significantly increase the risk of extubation failure [2,3,4]. Due to the limitations of anatomical and physiological changes in patients and other conditions during extubation, anesthetists may encounter more challenging situations than intubation [5]. Extubation failure and extubation-related complications after general anesthesia are significantly associated with morbidity and mortality. According to the findings of the Fourth National Audit Project of the Royal College of Anesthetists and the Difficult Airway Society, nearly 1/3 of the major airway complications occur in the extubation period or recovery room under general anesthesia, with a mortality rate of 5% [6], which shows the importance of improving airway management after general anesthesia. In the latest 2022 American Society of Anesthesiologists (ASA) Practice Guidelines for Management of the Difficult Airway, difficult or failed tracheal extubation was included in the definition of difficult airway for the first time [7], providing strong suggestions for difficult airway after surgery. However, no other specific tools or procedures can increase the safety of extubation for difficult airways, which has been widely recognized or routinely used in clinical practice [2,8,9,10], except for the use of an airway exchange catheter tube that can guide and accelerate reintubation [7,11,12,13,14].

Numerous clinical studies have been conducted on the application of machine-learning to the clinical decision-making process. The prediction model established using machine-learning could provide clinicians with an early warning before clinical symptoms occur. Based on mathematical and statistical methods, machine-learning models can be used to analyze and infer relationships between clinical signs and patient outcomes [15,16,17,18].

Currently, for patients with difficult airway after general anesthesia, there has been no exact risk factor analysis and prediction formula or model that can guide clinicians to prevent the occurrence of extubation failure. The purpose of this study was to develop a prediction model using clinical and laboratory machine-learning variables to predict extubation failure in adult patients undergoing head, neck, and maxillofacial surgeries under general anesthesia. This model will be helpful for clinicians to identify the risk of extubation failure in advance after administration of general anesthesia, thus reducing the morbidity and mortality of these patients.

## 2. Materials and Methods

All the data in this retrospective study were obtained from the inpatient and anesthesia systems of a single center. The study was approved by the ethics committee of the center (ethics no.: SH9H-2022-T234-1) and followed the transparent report of the multivariate prediction model used for individual prognosis or diagnosis, namely, the development and validation of the prediction model under the guidance of the principle of “TRIPOD” [19]. The requirement for informed consent was waived owing to the retrospective nature of the study.

### 2.1. Study Population and Design

A single-center retrospective study was conducted in adult patients who underwent head, neck, and maxillofacial general anesthesia between July 2015 and July 2022 at the Shanghai Ninth People’s Hospital. The main outcome of this study was extubation failure after general anesthesia, which was defined as the need for reintubation or emergency airway opening due to airway obstruction that could not maintain normal ventilation within 72 h after removal of the endotracheal tube after general anesthesia [2,20].

The following inclusion criteria were adopted for the case group: (1) general anesthesia patients who underwent head, neck, and maxillofacial surgeries from 1 July 2015 to 31 July 2021; (2) age ≥ 18 years; and (3) extubation failure after general anesthesia. The exclusion criteria were as follows: (1) preoperative tracheotomy or postoperative preventive tracheotomy; (2) occurrence of extubation failure, but not due to a difficult airway; and (3) the patient died from various causes before tracheal extubation. If a patient had multiple extubation failures and repeated catheterization, only the data from the first extubation failure were used.

**Figure 1:**
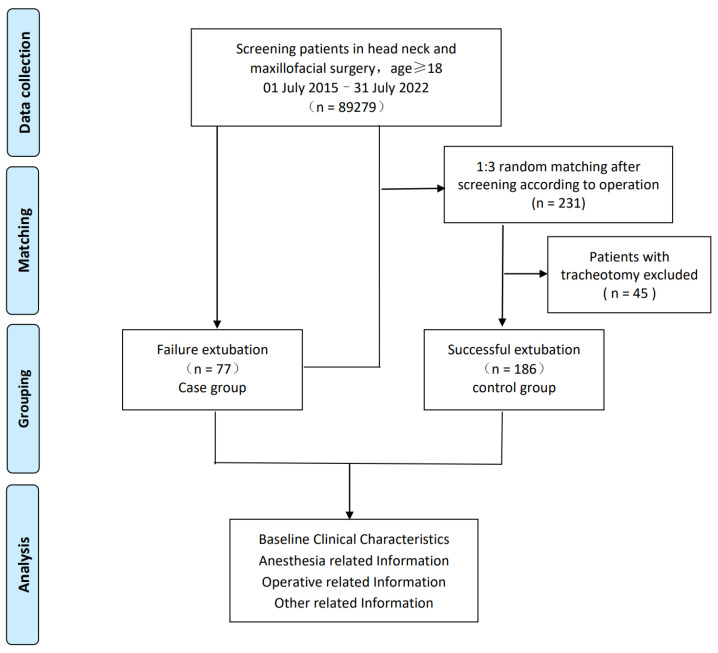
Flow chart of the case–control study.

Patients in the control group without extubation failure were selected from the above population. Considering that patients with a tracheotomy were excluded, we screened them according to the operative method of the case group and randomly matched them in a 1:3 ratio first and then excluded those who underwent a tracheotomy after surgery (shown in Figure 1).

### 2.2. Data Collection and Data Expansion

We extracted real inpatient data of patients who underwent head, neck, and maxillofacial surgeries from the hospital inpatient information system and anesthesia information system for the last 7 years. Patient characteristics and clinical data that might be related to extubation failure, which included baseline clinical characteristics, anesthesia-related information, operation-related information, and other related information, were collected (Table 1). The data used for the variable screens are presented in Supplementary Table S1.

The patients’ basic data (such as gender and age) and related medical history (such as disease history, treatment history, and surgical plan) as well as related physical examination and laboratory examination results (such as mouth opening, tumor size, and hemoglobin) were directly extracted from the patient inpatient information system. Data related to surgery and anesthesia (such as induction of anesthesia, operation time, and blood loss) was extracted directly from the anesthesia system. The specific definitions of ASA grade and surgical complexity grade are shown in Supplementary Tables S2 and S3, respectively.

The surgical compositions of the patients in the case and control groups are shown in Supplementary Table S4.

Low-quality data with missing values greater than 20% were excluded. For missing data, we used R packets for multivariate interpolation via the chain equation (MICE).

**Figure 2:**
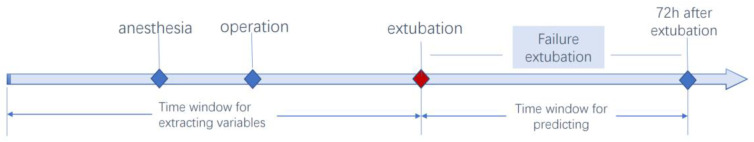
Data extraction and model prediction.

The time windows for the extraction of clinical variables were pre-anesthesia, intraoperative, and postoperative extubation. The time window of the prediction model was from the beginning of extubation to 72 h after extubation (Figure 2).

The bias in the number of cases between the two groups was corrected by extending the data. Data expansion was performed using the “ROSE” package in the R software. The data cases used for training were extended to match the photographic pairs. No data were extended to the test data.

### 2.3. Model Selection

Patient characteristics and clinical data were compared between the case and control groups, and logistic stepwise regression was performed to screen key features. Six machine-learning methods—random forest (RF), k-nearest neighbor (KNN), logistic regression (LOG), support vector machine (SVM), extreme gradient boosting (XGB), and light gradient boosting machine (GBM)—were selected to train models. The datasets were first randomly divided into training (70%) and test sets (30%). Five-fold cross-validation was performed on 70% of the training set to reduce the bias caused by randomly dividing the dataset. The models on each dataset were trained and evaluated, and the area under the curve (AUC) was calculated. Models with better results were selected and validated in 30% of the samples. We calculated the threshold-dependent measures of sensitivity, specificity, and accuracy for all models under the “best” threshold (Youden’s index). For the training set, 25 modeling sessions were conducted for each model (five multiple interpolation datasets) × 5 (k-fold), and the receiver operating characteristic (ROC) curve and other parameters of the median AUC were shown in the results. For the test set, each model was verified five times (five multiple interpolation datasets). Among them, the median ROC of each test followed by other parameters is also shown in the results.

### 2.4. Statistical Analysis

Data are reported as mean ± standard deviation, median (P25–P75), or percentage, depending on the type. The hypothesis was tested using a one-way analysis of variance, the Mann–Whitney *U* test, and Fisher’s exact probability method. A stepwise logical model was constructed with R. All six models were completed in R. Random forest used the “randomForest” package; SVM, the “e1071” package; KNN, the “kknn” package; XGB, the “xgboost” package; and GBM, the “lightgbm” package. The logistic regression used R’s default command: glm(). The median AUC was used to evaluate the effectiveness of the models and the ROC curve was displayed as the result of each model. Multiple interpolations were performed using the “MICE” package in R and the ROC curve was plotted using the pROC package in R 4.0.4. The confidence intervals (CI) of AUC were obtained using the bootstrapping method.

## 3. Results

A total of 89,279 adult patients who underwent head, neck, and maxillofacial surgeries in the past seven years were reviewed, and 77 patients had extubation failure. We screened and randomly matched at 1:3 according to the operative method of the case group and then excluded 60 patients who underwent tracheotomy after surgery; 186 patients with successful extubation were selected as the control group (Figure 1).

### 3.1. Baseline Variable Details of the Prediction Model

**Figure 3:**
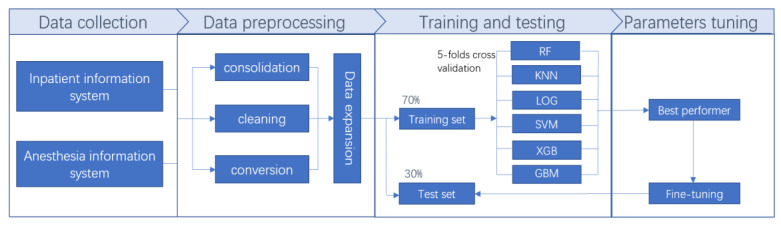
Flow diagram of the study.

Baseline patient clinical features and surgery-related and anesthesia-related clinical information applied to the prediction model are summarized in Table 1. The training set contained 174 cases and the test set contained 89 cases. Extubation failure occurred in 49 cases (28.1%) in the training set and 28 cases (31.4%) in the test set. The research process is shown in Figure 3.

### 3.2. AUC

We used stepwise logistic regression to select seven key variables: ASA classification, diabetes, neck radiotherapy history, maxillofacial surgery history, intraoperative bleeding, anemia, and hypokalemia before extubation from 26 candidate variables for further study, as shown in Table S2.

After training, the AUCs using RF, KNN, LOG, SVM, XGB, and GBM were 0.62 (95% CI, 0.40–0.83), 0.61 (95% CI, 0.38–0.84), 0.71 (95% CI, 0.52–0.89), 0.74 (95% CI, 0.55–0.93), 0.57 (95% CI, 0.40–0.74), and 0.67 (95% CI, 0.50–0.84), respectively.

**Figure 4:**
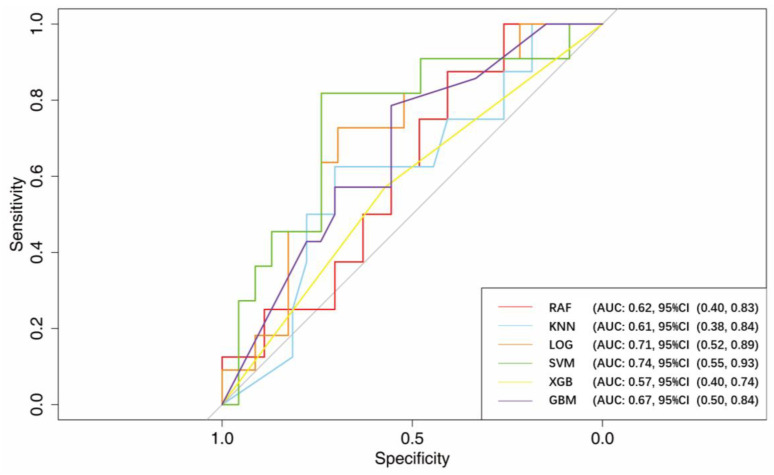
ROC curve of six machine-learning methods in k-folding set. A reference line indicated the AUC of 0.5.

The performance of the six models at the best thresholds is shown in Figure 4 and Table 2.

### 3.3. Optimal Model of Machine-Learning

After training, the LOG and SVM performed well. The AUC of LOG was 0.71, (95% CI, 0.52–0.89), and the AUC of SVM was 0.74 (95% CI, 0.55–0.93).

**Figure 5:**
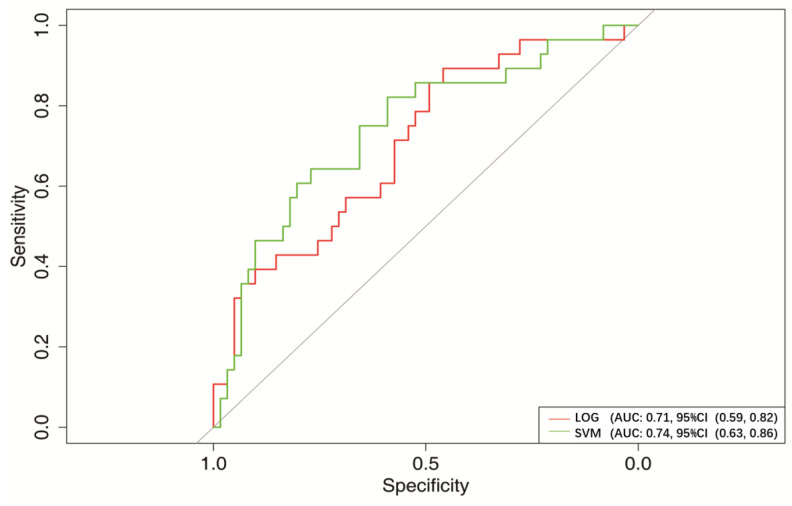
ROC curves of the two best models in the test set. A reference line indicated the AUC of 0.5.

Therefore, we verified these two models. In the verification set, the AUCs of LOG and SVM were 0.71 (95% CI, 0.59–0.82) and 0.74 (95% CI, 0.63–0.86), respectively (Figure 5 and Table 3).

### 3.4. Prediction of the Risk of Extubation Failure

**Figure 6:**
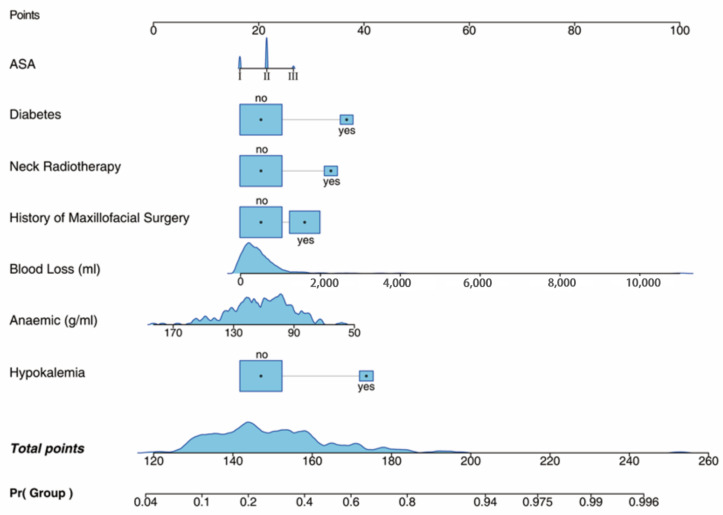
Assessment of the risk of extubation failure by logistic regression.

The two best models, namely, LOG and SVM, were further analyzed. We constructed logistic multifactor regression, analyzed the contribution degree of each influencing factor in the model to the regression coefficient of extubation failure, and drew a graph to predict the risk of extubation failure, which is more convenient to evaluate the extubation risk of patients after general anesthesia, as shown in Figure 6.

**Figure 7:**
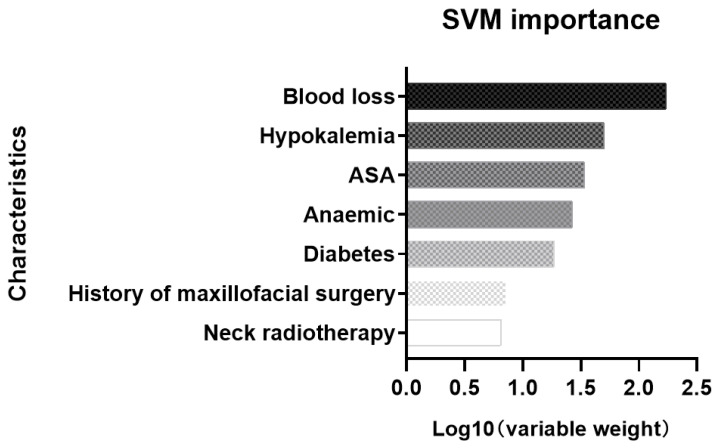
Feature importance of SVM.

For SVM, the importance of different variables in predicting extubation failure is shown in Figure 7.

## 4. Discussion

In this study, 89,279 patients who underwent general anesthesia for head, neck, and maxillofacial surgeries over seven years were reviewed. Extubation failure occurred in 77 patients. Clinical data obtained from case control studies were used to introduce six machine-learning methods: RF, KNN, LOG, SVM, XGB, and GBM. Finally, two optimal machine-learning models, LOG and SVM, were used to establish a multivariate prediction model of extubation failure in patients with a difficult airway after head, neck, and maxillofacial surgery.

Extubation failure after general anesthesia is significantly associated with morbidity and mortality. This retrospective study was conducted on patients who underwent head, neck, and maxillofacial surgeries, a high-risk population with a difficult airway after surgery. The clinical data of patients with extubation failure after general anesthesia in the past seven years were analyzed and compared with those of patients with successful extubation. To the best of our knowledge, this is the first time that a multivariate prediction model was established to provide clinicians with relatively objective indicators and a basis to reduce the risk of postoperative extubation failure in patients with difficult airways.

The mechanisms and risk factors that may increase the risk of patients with difficult airways after general anesthesia of head, neck, and maxillofacial surgeries are complex and diverse [2,5,20,21,22]. The final choice of postoperative airway management is usually made by clinicians according to their subjective experience, which may increase the incidence of postoperative airway complications. In recent years, predictive modeling using machine-learning algorithms has been widely used in clinical research, including the prediction of morbidity and mortality [23,24,25,26,27], in the field of critical care and pain medicine [17,28,29]. It has also been used in perioperative anesthesia management, including the prediction of the risk of induced low blood pressure and ventilator weaning [15,16,30,31]. Compared with other traditional algorithms, machine-learning has more advantages and greater flexibility in model-fitting. With the help of machine-learning, more accurate models can be developed for clinical diagnosis, prediction, and decision-making [26,32,33].

Finally, we screened out seven key variables to establish the model, and then analyzed and evaluated their risk level for extubation failure. Whether these key variables are appropriate for assessing the risk of postoperative extubation failure in patients with difficult airways, we found that most of them are in line with clinical practice and are supported by the literature. The first variable was the ASA classification, which is a commonly used clinical index to evaluate postoperative complications and anesthesia risk in patients and is widely used for risk prediction and many other purposes [34]. The second variable was a history of neck radiation. Radiation fibrosis syndrome is caused by radiotherapy of the head and neck; therefore, neck mobility and mouth opening are limited, which is related to the increased risk of difficult airways [35]. The third variable was a history of maxillofacial surgery. Changes in maxillofacial anatomy and defects in normal structures and tissues caused by maxillofacial surgery are also risk factors for a difficult airway [36]. The fourth and fifth variables were surgical blood loss and anemia before extubation. The maxillofacial surgical area was adjacent to the upper respiratory tract. Bleeding and swelling in the surgical area may increase the risk of airway obstruction, which is also an important factor for the increased risk of a difficult airway after head and neck surgery [1]. All of these five variables are directly related to the occurrence of a postoperative difficult airway. Thus, they are included in the prediction model by machine-learning.

Two other variables, namely, history of diabetes and hypokalemia before extubation, which did not appear to be directly related to extubation failure, were also included in the model. Diabetes and hypokalemia are correlated in clinical practice. We do not think that they are closely related to extubation failure after general anesthesia. Both are included in the model. In contrast, for diseases of the oral and maxillofacial region, changes in anatomy are speculated to lead to restricted mouth opening and difficulty in chewing and swallowing, which often result in an abnormal diet. The more severe the maxillofacial primary disease, the greater the influence on feeding and the more complicated the operation, both of which can cause severe pre- and postoperative hypokalemia. By contrast, severe hypokalemia is associated with serious adverse consequences, including myasthenia, arrhythmia, and death [37]. Reintubation after planned extubation after elective surgery is more common in critically ill patients (up to 0.4–25%) [11]. Diabetes is also a risk factor for increased postoperative complications. Finally, hypokalemia may further aggravate the injury or disappearance of protective reflexes such as swallowing and coughing, caused by tissue edema and nerve injury after head, neck, and maxillofacial surgeries. All of these factors may increase the risk of extubation failure. Further research is warranted to confirm these assumptions.

Notably, the key variables screened herein were frequently used in clinical practice and relatively cheap, simple, and easy to measure. Such a model undoubtedly has great practical utility and is easy to apply in clinical practice.

This study has several limitations. First, as this was a retrospective study, some missing clinical characteristic parameters are inevitable. Although we have supplemented the missing data, we might have improved the prediction performance of the machine-learning model if we could collect more complete data. Second, all the patients included in this study were Chinese; thus, extending the machine-learning model and the new calculation formula developed in this study to other countries is difficult. Finally, the AUC of the model could not reach a satisfactory degree. This may need to be remedied by obtaining better and more accurate prospective test data.

In conclusion, our study established a multivariable prediction model to predict extubation failure in patients with difficult airways after general anesthesia of head, neck, and maxillofacial surgeries. The key indicators used in the model are simple, easy to obtain, and worthy of clinical application. This will help to reduce the morbidity and mortality of these patients after general anesthesia.

## Figures and Tables

**Table 1:** Baseline patient clinical features and surgical- and anesthesia-related clinical information applied to the prediction model.

Characteristic	Case	Control	*p*
	n = 77	n = 186	
Baseline Clinical Characteristics			
Sex			
Male (%)	53 (68.8)	118 (63.4)	0.478
Female (%)	24 (31.2)	68 (36.6)	
Age (year)	60 (48, 71)	59 (41, 68)	0.539
Surgical complexity			
I (%)	1 (1.3)	0 (0.0)	0.061
II (%)	4 (5.2)	19 (10.2)	
III (%)	17 (22.1)	58 (31.2)	
IV (%)	55 (71.4)	109 (58.6)	
ASA grade			
I (%)	10 (13.2)	61 (32.8)	0.003
II (%)	61 (80.3)	116 (62.4)	
III (%)	5 (6.6)	9 (4.8)	
COPD			
No (%)	74 (96.1)	179 (96.2)	1
Yes (%)	3 (3.9)	7 (3.8)	
Hypertension			
No (%)	57 (74.0)	144 (77.4)	0.632
Yes (%)	20 (26.0)	42 (22.6)	
Diabetes			
No (%)	66 (85.7)	173 (93.0)	0.097
Yes (%)	11 (14.3)	13 (7.0)	
OSAS			
No (%)	75 (98.7)	180 (96.8)	0.677
Yes (%)	1 (1.3)	6 (3.2)	
Coronary heart disease			
No (%)	72 (93.5)	180 (96.8)	0.308
Yes (%)	5 (6.5)	6 (3.2)	
Anesthesia-related Information			
Induction			
Anesthetized (%)	58 (76.3)	157 (84.4)	0.155
Awake (%)	18 (23.7)	29 (15.6)	
Mouth opening (cm)	4.00 (3.00, 4.00)	4.00 (3.50, 4.00)	0.697
History of neck radiotherapy			
No (%)	63 (82.9)	176 (94.6)	0.007
Yes (%)	13 (17.1)	10 (5.4)	
History of maxillofacial surgery			
No (%)	38 (50.0)	138 (74.2)	<0.001
Yes (%)	38 (50.0)	48 (25.8)	
Operation-related Information			
Tumor size (cm)	2.80 (0.00, 4.80)	2.00 (0.35, 3.12)	0.103
Operation time (h)	5.58 (3.75, 8.50)	4.75 (2.75, 6.75)	0.003
End time (24 h)	16 (14, 19)	16 (14, 18)	0.444
Blood loss (mL)	400 (300, 763)	300 (100, 500)	<0.001
Blood infusion (mL)	0 (0, 500)	0 (0, 0)	0.032
Fluid infusion (mL)	3400 (2000, 4000)	2500 (1500, 3500)	0.002
Surgical site			
Intraoral	17 (22.1)	43 (23.1)	0.995
Neck (%)	12 (15.6)	29 (15.6)	
Skull base (%)	20 (26.0)	45 (24.2)	
≥2 sites (%)	28 (36.4)	69 (37.1)	
Flap reconstruction			
No (%)	44 (57.9)	133 (71.5)	0.042
Yes (%)	32 (42.1)	53 (28.5)	
Other Related Information			
Extubation time (h)	14.50 (6.00, 37.00)	15.75 (1.00, 39.69)	0.403
Delirium			
No (%)	73 (94.8)	170 (91.4)	0.448
Yes (%)	4 (5.2)	16 (8.6)	
Anemia (g/L)	104 (95, 115)	116 (100, 130)	<0.001
Hypokalemia			
No (%)	62 (80.5)	164 (94.8)	<0.001
Yes (%)	15 (19.5)	9 (5.2)	

ASA, American Society of Anesthesiologists; COPD, chronic obstructive pulmonary disease; OSAS, obstructive sleep apnea syndrome.

**Table 2:** Performance of the six models at the best threshold in the k-fold set.

Variables	AUC (95% CI)	Specificity (95% CI)	Sensitivity (95% CI)	Accuracy (95% CI)
RF	0.62 (0.40–0.83)	0.48 (0.19–1.00)	1.00 (0.38–1.00)	0.57 (0.37–0.86)
KNN	0.61 (0.38–0.84)	0.70 (0.15–0.89)	0.75 (0.38–1.00)	0.71 (0.34–0.86)
LOG	0.71 (0.52–0.89)	0.65 (0.30–0.91)	0.82 (0.45–1.00)	0.71 (0.53–0.85)
SVM	0.74 (0.55–0.93)	0.74 (0.52–0.96)	0.82 (0.55–1.00)	0.76 (0.62–0.91)
XGB	0.57 (0.40–0.74)	0.57 (0.00–1.00)	0.57 (0.00–1.00)	0.60 (0.40–0.74)
GBM	0.67 (0.50- 0.84)	0.63 (0.19–0.89)	0.79 (0.43–1.00)	0.68 (0.46–0.80)

AUC, area under the curve; CI, confidence interval; GBM, light gradient Boosting Machine; KNN, K-nearest neighbor; LOG, logistic regression; RAF, random forest; SVM, support vector machine; XGB, extreme Gradient Boosting.

**Table 3:** Performance of the two best models in the test set.

Variables	AUC (95% CI)	Specificity (95% CI)	Sensitivity (95% CI)	Accuracy (95% CI)
LOG	0.71 (0.59, 0.82)	0.56 (0.36, 0.97)	0.86 (0.36, 1.00)	0.66 (0.54, 0.82)
SVM	0.74 (0.63, 0.86)	0.75 (0.49, 0.95)	0.75 (0.46, 0.96)	0.74 (0.61, 0.84)

## Data Availability

The data that support the findings of this study are available from the corresponding author upon reasonable request (email: zhouren77@126.com).

## Supplementary Materials

The following supporting information can be downloaded at: https://www.mdpi.com/article/10.3390/jcm12031066/s1, Table S1: Patient operation types in the case and control groups; Table S2: Model application variables and final included variables; Table S3: Surgery complexity Grades; Table S4: Patient operation types in the case and control groups.
