# Supplementary material for: Development of a Machine-Learning Model for Prediction of Extubation Failure in Patients with Difficult Airways after General Anesthesia of Head, Neck, and Maxillofacial Surgeries
Source: J Clin Med. 2023 Jan 30;12(3):1066. doi: 10.3390/jcm12031066 (PMC9917752; doi:10.3390/jcm12031066)
Supplement: Supplementary file 1 [file jcm-12-01066-s001.zip › jcm-2178968-supplementary.pdf]

Supplementary Materials

Table S1: Model application variables and final included variables

Performance characteristics of models for predicting failure extubation	Variables included into the model
Sex	ASA
Age (year)	History of neck radiotherapy
Surgical complexity	Diabetes
ASA	History of maxillofacial surgery
COPD	Blood loss(ml)
Hypertension	Anemia
Diabetes	Hypokalemia
OSAS	
Coronary heart disease	
Anesthesia induction	
Mouth opening (cm)	
History of neck radiotherapy	
History of maxillofacial surgery	
Tumor size (cm)	
Operation time (h)	
End time of operation (24 h)	
Blood loss (mL)	
Blood infusion (mL)	
Fluid infusion (mL)	
Surgical site	
Flap repair	
Extubation time (h)	
Anemia (g/L)	
Hypokalemia	

Table S2: ASA Physical Status Classification

ASA grade	Definition
I	Normal healthy patient
II	Patient with mild systemic disease
III	Patient with severe systemic disease
IV	Patient with severe systemic disease that is a constant threat to life

ASA , American Society of Anesthesiologists.

Table S3: Surgery complexity Grades

Surgery complexity Grades	Complexity	risk	Examples
Ⅰ	Minor	Low	Excising skin lesion, Curettage of cyst
Ⅱ	Intermediate	Moderate	Tonsillectomy or Adenotonsillectomy
Ⅲ	Complex	High	Thyroidectomy, BSSRO
Ⅳ	Major and Complex	High	Radical neck dissection, Flap reconstruction

Table S4: Patient operation types in the case and control groups

Surgical procedure	Case	Control	Tracheotomy excluded
Uvulopalatopharyngoplasty, UPPP	2	6	0
Extended resection of nasal tumor/flap reconstruction	2	6	0
Radical resection of buccal tumor/flap reconstruction	9	23	4
Excision of thyrohyoid cyst	2	6	0
Neck scar excision/flap reconstruction	1	3	0
Excision of carotid body tumor	1	3	0
Anterior cervical interbody fusion	3	9	0
Mandibular bilateral ramus sagittal split ramus osteotomy , BSSRO	1	3	0
Mandibulectomy/radical resection/flap reconstruction	8	15	9
Functional/radical neck nodes dissections	4	8	4
Radical resection of tongue/floor of mouth tumor/flap reconstruction	12	24	12
Excision of parapharyngeal tumor/flap reconstruction	1	1	2
Skull base tumor resection/flap reconstruction	13	31	8
Open reduction of condylar fracture	1	3	0
Reduction and internal fixation of temporomandibular joint	3	9	0
Parotidectomy/radical resection/flap reconstruction	5	11	4
Maxillectomy/radical resection/flap reconstruction	6	17	1
Excision of neurofibroma of head and neck	1	3	0
Combined dissection of gingiva and mandible and neck/flap reconstruction	2	5	1
Total	77	186	45
